# The Forkhead Transcription Factor, *Foxd1*, Is Necessary for Pituitary Luteinizing Hormone Expression in Mice

**DOI:** 10.1371/journal.pone.0052156

**Published:** 2012-12-19

**Authors:** Jason H. Gumbel, Elizabeth M. Patterson, Sarah A. Owusu, Brock E. Kabat, Deborah O. Jung, Jasmine Simmons, Torin Hopkins, Buffy S. Ellsworth

**Affiliations:** Department of Physiology, Southern Illinois University, Carbondale, Illinois, United States of America; Baylor College of Medicine, United States of America

## Abstract

The pituitary gland regulates numerous physiological functions including growth, reproduction, temperature and metabolic homeostasis, lactation, and response to stress. Pituitary organogenesis is dependent on signaling factors that are produced in and around the developing pituitary. The studies described in this report reveal that the forkhead transcription factor, *Foxd1*, is not expressed in the developing mouse pituitary gland, but rather in the mesenchyme surrounding the pituitary gland, which is an essential source of signaling factors that regulate pituitary organogenesis. Loss of *Foxd1* causes a morphological defect in which the anterior lobe of the pituitary gland protrudes through the cartilage plate that is developing ventral to the pituitary at embryonic days (e)14.5, e16.5, and e18.5. The number of proliferating pituitary cells is increased at e14.5 and e16.5. Loss of *Foxd1* also results in significantly decreased levels of *Lhb* expression at e18.5. This decrease in *Lhb* expression does not appear to be due to a change in the number of gonadotrope cells in the pituitary gland. Previous studies have shown that loss of the LIM homeodomain factor, *Lhx3*, which is activated by the FGF signaling pathway, results in loss of LH production. Although there is a difference in *Lhb* expression in *Foxd1* null mice, the expression pattern of LHX3 is not altered in *Foxd1* null mice. These studies suggest that *Foxd1* is indirectly required for normal *Lhb* expression and cartilage formation.

## Introduction

The pituitary gland is a highly specialized organ that is essential for normal endocrine function. This essential gland secretes hormones that regulate growth, metabolism, reproduction, lactation, and response to stress [1]. Pituitary organogenesis begins in mice on embryonic day (e)8.5. By e10.5 the oral ectoderm invaginates and will form Rathke’s pouch by e12.5. The early stages of pituitary development are characterized by rapid proliferation. This is evident at e14.5 by the significant expansion of the anterior lobe of the pituitary. The last day of mouse embryonic development is e18.5 (the day before birth) [2].

The anterior lobe of the pituitary gland contains five specialized hormone-secreting cell types. Somatotropes produce growth hormone (GH) that targets the liver and bone. Lactotropes secrete prolactin (PRL) that acts on the mammary glands. Gonadotropes produce follicle-stimulating hormone (FSH) and luteinizing hormone (LH) that regulate function of the gonads. Thyrotropes secrete thyroid-stimulating hormone (TSH) that targets the thyroid. FSH, LH, and TSH are dimeric hormones consisting of a common α-subunit (CGA) and a unique β-subunit (FSHB, LHB, TSHB). Finally, corticotropes produce adrenocorticotropic hormone (ACTH) that acts on the adrenal gland. The posterior lobe of the pituitary gland is stimulated by direct innervation from the hypothalamus and secretes oxytocin and anti-diuretic hormone. The intermediate lobe produces melanocyte-stimulating hormone. Input from the hypothalamus stimulates the pituitary to secrete hormones that act on a number of target organs throughout the body to regulate a diverse range of physiological functions [2].

Differentiation of the different cell types is dependent on dorsal-ventral morphogenetic gradients that result in overlapping dorsal-ventral patterns of transcription factor expression. Signaling molecules such as bone morphogenetic proteins (BMPs), fibroblast growth factors (FGFs), and sonic hedgehog (SHH) are involved in initiating pituitary development [3], [4].

Several forkhead factors have roles in pituitary development and function. *Foxl2* (*Pfrk*) is the first forkhead to be described in the pituitary gland [5]. FOXL2 protein is detected in the prospective anterior lobe of the developing pituitary gland starting at e11.5 and continuing into adulthood in gonadotrope and thyrotrope cells of the anterior pituitary [6]. FOXL2 plays a role in regulating several gonadotropin genes including those coding for gonadotropin-releasing hormone receptor, the glycoprotein hormone α-subunit (*Cga*), and *Fshb* [6], [7], [8], [9], [10]. In fact, expression of *Fshb* is severely impaired in the absence of *Foxl2*, suggesting that *Foxl2* is required for normal *Fshb* expression [10]. The forkhead factor, FOXP3, has a well-established role in the development and function of helper T cells [11], [12]. While *Foxp3* is not expressed in or even near the pituitary gland, it is important for pituitary function [13]. *Scurfy* mice have a mutation in *Foxp3* and have drastically reduced levels of *Lhb* and *Fshb* expression resulting in infertility [13], [14], [15]. FOXA1 represses growth hormone expression in mouse and human pituitary [16]. In cell culture studies with a gonadotrope-derived cell line, FOXO1 represses expression of *Lhb* [17]. *Foxf1* is expressed in the mesenchyme surrounding the developing pituitary gland and in the adult posterior and anterior pituitary [18]. Finally, *Foxe1* is expressed in Rathke’s pouch from e10.5–e11.5, however pituitary hormones are normal in *Foxe1* null pups [19].

*Foxd1* was originally known as brain factor-2 (*Bf2*) and is important for proper kidney formation [20], [21]. Heterozygous null mice have no obvious phenotype and are fertile [20]. *Foxd1* homozygous null mice have small kidneys, decreased ureteric branching and die within 24 hours after birth due to renal failure. This is due, in part, to ectopic bone morphogenetic (BMP) signaling, which causes mis-patterning of the kidney [20]. *Foxd1* is also expressed in the retina and is required for normal development of the retina and optic chiasm [22]. The following studies demonstrate that while *Foxd1* is not expressed in the developing pituitary gland, it is expressed in the mesenchyme surrounding the pituitary gland, which produces signaling factors that are essential for normal pituitary development.

## Materials and Methods

### Mice

*Foxd1* heterozygous null mice were generated by Hatini et al. [20] and provided to us by Cathy Mendelsohn of Columbia University. To create the null *Foxd1* allele, a *LacZ* cassette was inserted in place of the *Foxd1* coding sequence. A phosphoglycerokinase (PGK)-*neo^r^* cassette was inserted in the same orientation [20]. As *Foxd1* is a single exon gene, this procedure eliminated nearly all of the *Foxd1* coding sequence. Mice were maintained in a 12-hour dark-light cycle. Embryos were obtained from an intercross of *Foxd1^LacZ/+^* mice. *Foxd1* mutant mice are on a mixed Swiss Webster and C57BL/6J background. The morning the copulatory plug is detected is determined to be e0.5. Littermates were used for all experiments in which normal and mutant embryos were compared. Live mice were genotyped using PCR with primers that amplify a region of the *neo^r^* gene (5′- ACCTTGCTCCTGCCGAGAAAGTAT and 5′- ATGTTTCGCTTGGTGGTCGAATGG). Embryos were genotyped by PCR with the following primers: 5′ - TCC CTT TAG CCC GGT TAG TCC AGG, 5′ - GCT CTG ACG TGC ACA CCA TGT GAC AG, 5′ - ATT CAG GCT GCG CAA CTG TTG GGA with DMSO and hot start.

The Southern Illinois University Animal Care and Use Committee approved all procedures using mice (Protocol Number: 10-020). All experiments were conducted in accord with the principles and procedures outlined in the NIH Guidelines for the Care and Use of Experimental Animals.

### β-galactosidase Staining

*Foxd1^LacZ/+^* embryos (e12.5-e18.5) were frozen and cryo-sectioned (5 µm). Frozen sections were post-fixed in 4% formaldehyde for 5 min, rinsed in PBS and stained overnight in β-galacotosidase staining solution in 1X PBS (5 mM potassium ferricyanide, 5 mM potassium ferrocyanide, 1 mg/mL X-gal). *Foxd1^LacZ/+^* embryos (e10.5) and adult pituitaries were stained whole mount for β-galactosidase as follows. Embryos and adult pituitaries were fixed in 4% formaldehyde for 1 hour, rinsed in PBS and stained overnight in β-galactosidase staining solution. After a series of graded ethanol washes, samples were embedded in paraffin and sectioned (5 µm).

### Histology and Immunohistochemistry

Embryos were dissected and fixed in 4% paraformaldehyde in phosphate buffered saline (PBS) (pH 7.2) for 45 min to 24 h (depending on stage of development). All samples were washed in PBS, dehydrated in a graded series of ethanol, and embedded in paraffin. Sections (5 µm) were deparaffinized in xylene, rehydrated through a series of graded ethanol washes, and stained in hematoxylin (Fisher Scientific) and eosin (Sigma) or used for immunohistochemistry.

To detect cell proliferation in embryonic pituitaries, pregnant mice were given an intraperitoneal injection of bromodeoxyuridine (BrdU) at 100 µg/g body weight 2 h before the embryos were harvested [23].

To visualize BrdU and LHX3, tissue sections were deparaffinized in xylene, rehydrated in ethanol, and 1.5% peroxide in water was used to remove endogenous peroxidases. After epitopes were unmasked by boiling in 10 mM citric acid for 10 min, tissue sections were blocked using the Mouse on Mouse (M.O.M.) kit (Vector Laboratories) according to the manufacturer's directions. Tissue sections were incubated overnight at 4°C with antibodies for BrdU (Invitrogen, clone ZBU30, 1∶100) or LHX3 (Developmental Studies Hybridoma bank, University of Iowa, 1∶1000). Tissue sections were incubated with anti-mouse secondary (MOM kit, Vector Laboratories) 30 min at room temperature. Next, sections were incubated sequentially with strept-avidin-horseradish peroxidase and fluorescein from the Tyramide Signal Amplification kit (PerkinElmer). Following a 5 min incubation with water, sections were counterstained with 4′,6-diamidino-2-phenylindole (DAPI) (167 nM, Molecular Probes). One mid-sagittal section from each individual was photographed at a magnification of 200X. The total number of BrdU-positive cells in the intermediate and anterior lobe of each section was counted manually in three individuals per group.

To visualize pituitary hormones, tissue sections were deparaffinized and rehydrated as described above. Tissue sections were incubated with antibodies against GH (1∶10,000; National Hormone and Peptide Program (NHPP)), POMC (1∶500; NHPP), TSHB (1∶2000, NHPP), LHB (1∶500, NHPP), CGA (1∶300), or FSHB (1∶250) for 1 hour at room temperature and then the appropriate secondary antibodies: anti-rabbit-TRITC (1∶100, Jackson ImmunoResearch), anti-guinea pig-FITC (1∶100, Jackson ImmunoResearch), or biotinylated anti-rabbit followed by horseradish peroxidase and FITC as described above. One mid-coronal section was photographed at a magnification of 200X for each individual. One half of the section was pictured in each photograph. Because coronal pituitary sections are symmetrical, this gives a good representation of cell types of the anterior lobe. The total number of LHB-positive cells in each photograph was counted manually. Three individuals per group were analyzed and values were set relative to wild type controls.

Programmed cell death in the pituitaries was also detected by the TUNEL method using the in situ cell detection kit POD (Roche) according to manufacturer’s instructions.

Cartilage was stained by Gomori’s aldehyde fuchsin stain as follows. Tissue sections were deparaffinized and rehydrated before incubating 10–60 min in 0.5% iodine. Tissue sections were then decolorized with 0.5% sodium bisulfite, washed in water and transferred to 70% alcohol. Tissue sections were stained in aldehyde fuchsin solution (0.5% basic fuchsin, 70% ethanol, 1% paraldehyde, pH 1.0) for 1–2 hr and rinsed in 70% ethanol.

Digital images of pituitary sections were captured with a Leica DM 5000B fluorescent microscope and Retiga 2000R digital camera. FITC and DAPI pictures were merged using Adobe Photoshop CS3.

### RT-PCR

Pituitaries were dissected from e18.5 embryos. Total RNA was isolated with the RNAqueous-Micro kit (Ambion, Inc.) according to manufacturer’s directions. RNA concentrations were determined by spectrophotometry. RNA was treated with DNase I and DNase inactivating reagent from the TURBO DNase-free kit (Ambion, Inc.) as per manufacturer’s instructions. ImPromII reagents and random primers (Promega) were used to synthesize cDNA.

Real time RT-PCR was performed on a CFX96 Real Time System (BioRad). Amplification was accomplished using Taqman Gene Expression Assays (Applied Biosystems) as per manufacturer’s instructions. Five ng of cDNA was used in a 15 µL reaction volume. Samples and controls were run in triplicate. No-template controls and no-reverse transcriptase controls were used to assure the absence of contamination and efficacy of the DNase treatment, respectively. Five to six individuals were included in each group. Data were analyzed by the ΔΔC_T_ method [24], [25]. The values for ΔΔC_T_ were calculated by subtracting the average ΔC_T_ of wild type controls from the ΔC_T_ for each sample. C_T_ values over 30 were considered unreliable and were not included in our analyses.

### Statistical Analysis

All results are expressed as mean ± SEM. Data were analyzed by Student’s t-test using Microsoft Excel. P-values less than 0.05 were considered significant (*). P-values less than 0.01 were considered very significant (**).

## Results

### Normal *Foxd1* Expression

**Figure 1:**
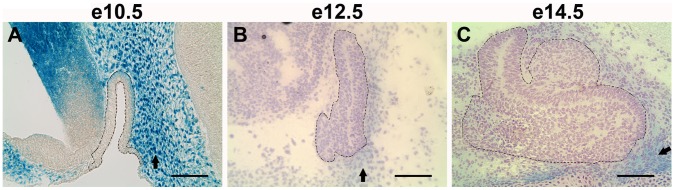
*Foxd1* is expressed in the mesenchyme surrounding the developing pituitary gland. X-gal staining of mid-sagittal sections from *Foxd1^+/LacZ^* embryos shows *Foxd1* expression (blue) in the mesenchyme (arrows) surrounding the developing pituitary gland at e10.5 (A), e12.5 (B), and e14.5 (C). *Foxd1* expression is not apparent in the pituitary gland itself (dotted lines) during development. Whole mount β-galactosidase staining was performed at e10.5 (A). At later ages, embryos were frozen and sections were stained for β-galactosidase (B, C). Pictures were taken at 200X and scale bars represent 100 µm.

*Foxd1* was identified in an expression library developed from pituitary at e14.5, suggesting that it may be important for pituitary development [26]. *Foxd1* expression was confirmed by real time RT-PCR (data not shown). To visualize *Foxd1* expression, mice in which the *Foxd1* allele has been replaced with the coding sequence for *LacZ* were analyzed [20]. Sections from mouse embryos that were heterozygous for the engineered *Foxd1* allele (*Foxd1^+/LacZ^*) were stained for β-galactosidase. This study reveals that *Foxd1* is not expressed in the pituitary gland during development, but rather in the mesenchyme surrounding the pituitary (Fig. 1A–C). Many signaling factors that are important for pituitary organogenesis are expressed in the mesenchyme surrounding the pituitary, thus FOXD1 could contribute to transcriptional regulation of these factors [27], [28].

### Pituitary Morphology

**Figure 2:**
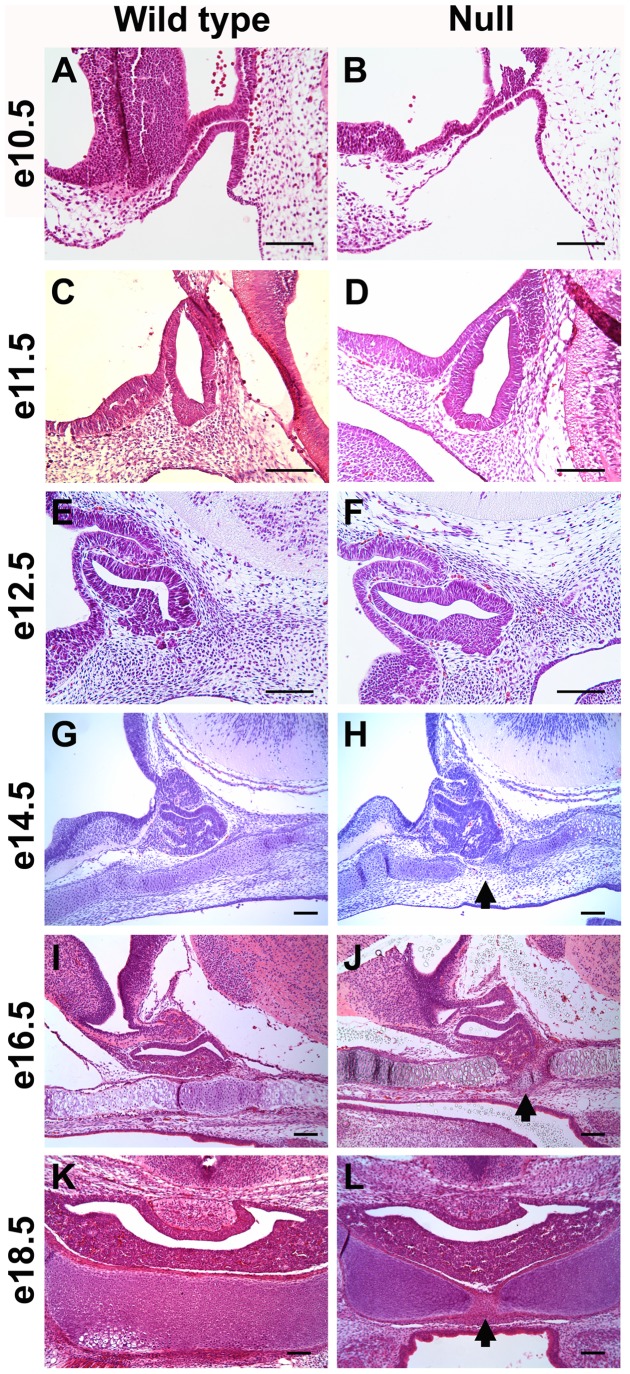
Loss of *Foxd1* expression results in pituitary dysmorphology. Hematoxylin and eosin staining of mid-sagittal sections (A–J) and coronal sections (K–L) revealed that the developing pituitary protrudes through the cartilage plate by e14.5 in *Foxd1^LacZ/LacZ^* embryos (arrows). Pictures were taken at 200X (A–F) or 100X (G–L). Scale bars represent 100 µm.

**Figure 3:**
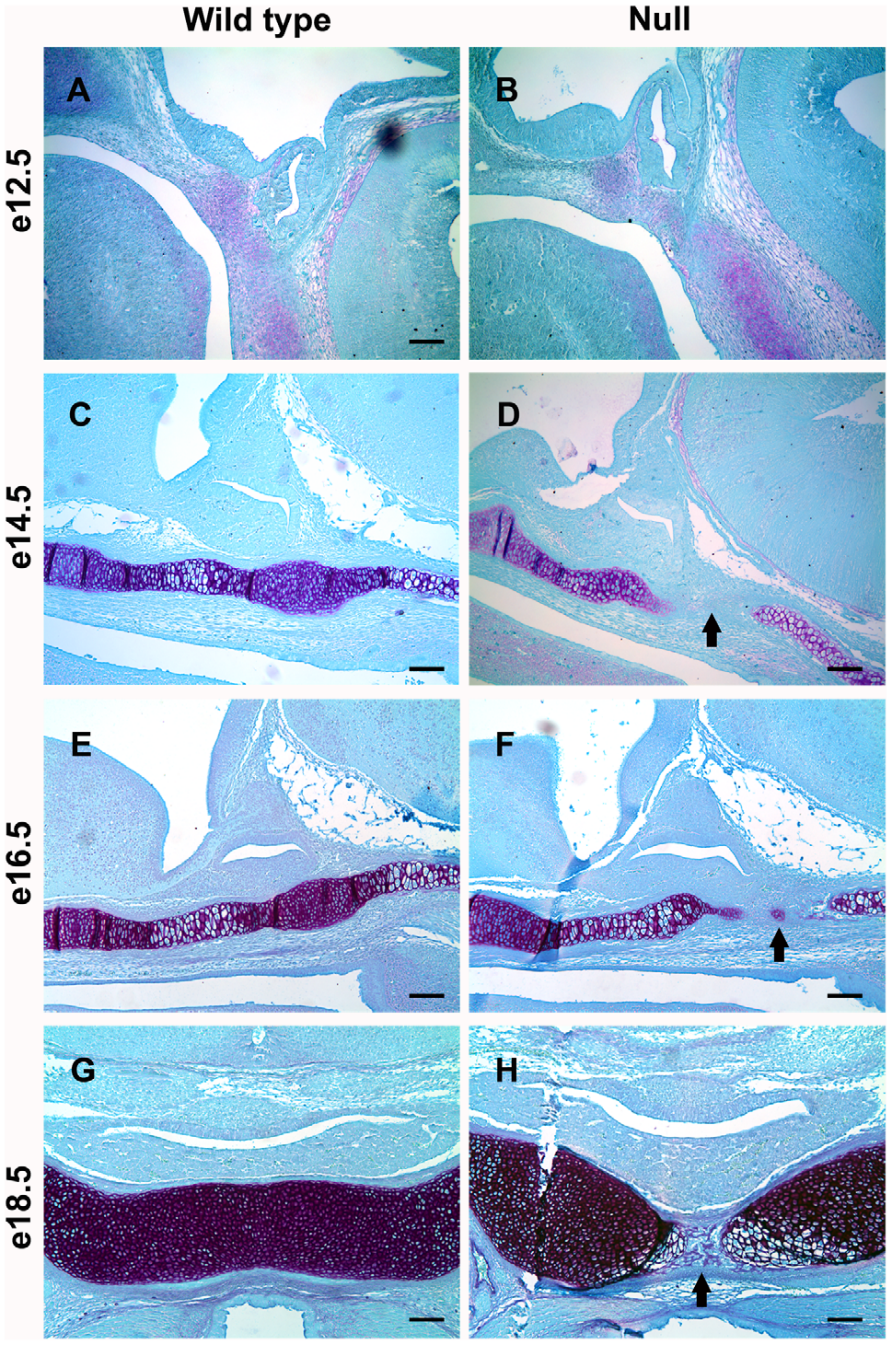
Loss of *Foxd1* expression results in disruption of the developing cartilage plate. Mid-sagittal (A–F) and coronal (G–H) sections from *Foxd1^LacZ/LacZ^* embryos and wild type littermates were stained with Gomori’s aldehyde fuchsin to visualize cartilage. (A–B) The prospective pituitary gland and surrounding tissue appears normal at e12.5. (C–H) A break in the cartilage plate ventral to the pituitary is apparent in *Foxd1^LacZ/LacZ^* embryos by e14.5 (arrows). Pictures were taken at 100X and scale bars represent 100 µm.

The expression of *Foxd1* in the mesenchyme surrounding the developing pituitary led us to investigate the role of FOXD1 in pituitary development. Morphology of the pituitary gland was examined in embryos lacking *Foxd1* expression. Hematoxylin and eosin stains of *Foxd1* null and wild type embryos revealed that the pituitary gland extends through the cartilage plate ventral to the pituitary gland (Fig. 2A–L). To better visualize the cartilage plate, Gomori’s aldehyde fuchsin stains were performed. These experiments showed that the developing pituitary extends through the cartilage plate evident by e14.5 (Fig. 3A–H).

### Pituitary Hormone Expression

**Figure 4:**
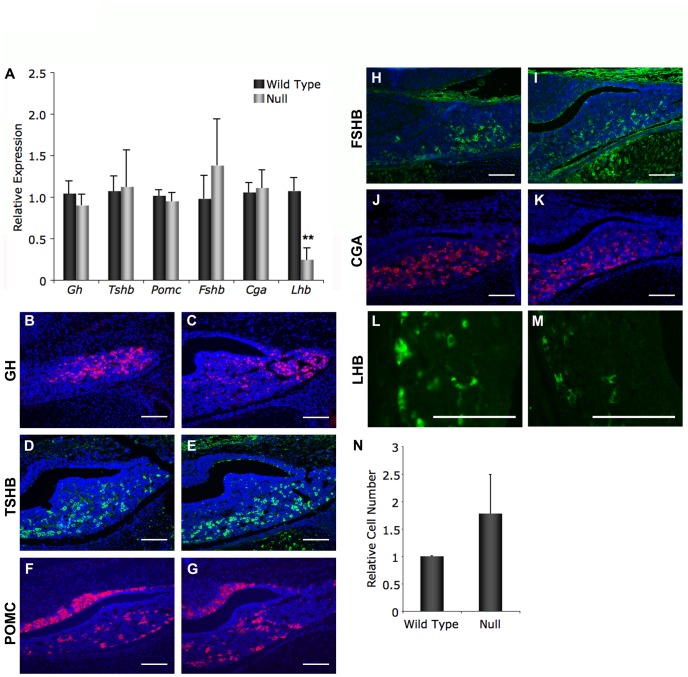
Expression of luteinizing hormone is reduced in *Foxd1^LacZ/LacZ^* embryos. (A) Real time RT-PCR revealed that expression of *Lhb* is significantly reduced in embryos lacking *Foxd1* expression compared to wild type littermates at e18.5 (P<0.05). Expression of other pituitary hormones was not significantly reduced. (B–K) No apparent differences in GH, TSHB, POMC, FSHB, or CGA were observed. (L–M) The intensity of LHB staining is reduced in mutant pituitary glands. (N) The number of LHB-positive cells was counted manually and set relative to wild type controls. No significant difference was detected in *Foxd1^LacZ/LacZ^* pituitaries as compared to wild type. Cell nuclei are stained with DAPI (blue). Pictures were taken at 200X (B–K) or 630X (L–M). Scale bars represent 100 µm.

The pituitary gland is essential for orchestrating numerous physiological processes. To determine if *Foxd1* expression is important for pituitary function, we performed real time RT-PCR on pituitary from e18.5 *Foxd1* wild type and null embryos. This age was chosen because most pituitary hormones are detectable at this age and *Foxd1* null mice die within 24 hours after birth. *Prl* was excluded from this analysis because C_T_ values for *Prl* at e18.5 are consistently over 30 and we feel that this does not represent an accurate measurement of expression (data not shown). This is consistent with findings by Brannick et al., demonstrating that neither prolactin protein nor prolactin mRNA is detectable until after postnatal day 1 [29]. Expression of growth hormone, thyroid-stimulating hormone-β, pro-opiomelanocortin, follicle-stimulating hormone-β, and the glycoprotein hormone α-subunit is not significantly different between wild type and null embryos (Fig. 4A). However, expression of luteinizing hormone is significantly decreased in *Foxd1* null embryos as compared to wild type littermates (Fig. 4A). No apparent loss of somatotrope, thyrotrope, or corticotrope cells was observed in *Foxd1* null embryos (Fig. 4B–K). While the intensity of staining is decreased for LHB, the number of gonadotrope cells is not decreased in *Foxd1* null embryos, suggesting that it is the level of *Lhb* expression that is decreased and not an inability of the gonadotropes to differentiate that causes the lower *Lhb* expression (Fig. 4L–N). This suggests that FOXD1 is required for normal pituitary expression of luteinizing hormone.

### Pituitary Cell Apoptosis and Proliferation

**Figure 5:**
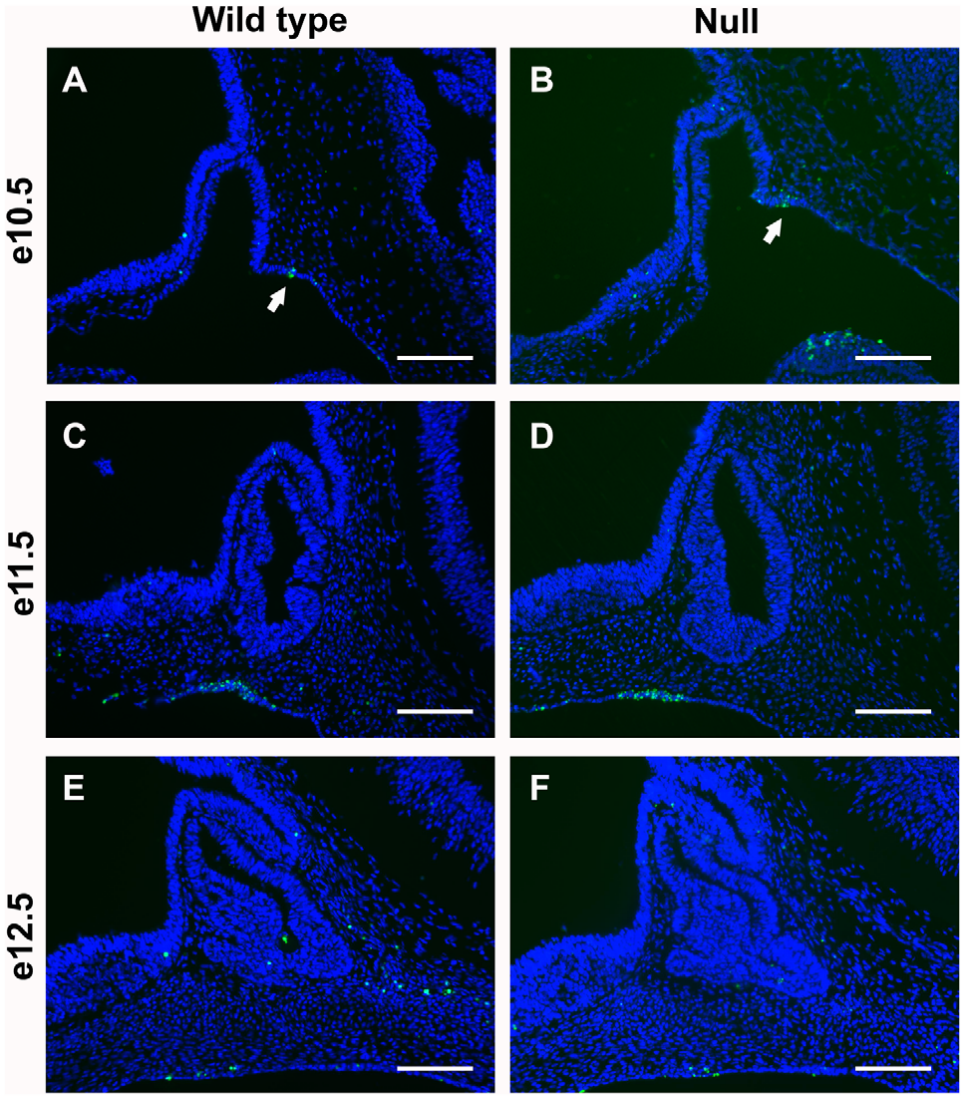
Apoptosis is not different in *Foxd1^LacZ/LacZ^* embryos. TUNEL was performed to label apoptotic cells in mid-sagittal sections from *Foxd1^LacZ/LacZ^* embryos and wild type littermates. (A–F) No loss of apoptosis was observed. (A–B) Arrows indicate regions of pituitary cell apoptosis. Pictures were taken at 200X and scale bars represent 100 µm.

Apoptosis and proliferation are essential processes for normal pituitary development. Apoptosis occurs at e10.5 to separate Rathke’s pouch from the oral ectoderm that will form the lining of the mouth [30]. After Rathke’s pouch is separated from the rest of the oral ectoderm, a cartilage plate forms to separate the pituitary from the oral cavity. If apoptosis fails to occur, the pituitary will remain attached to the rest of the oral ectoderm and the cartilage plate will not form completely [31]. To determine if failure of apoptosis caused the pituitary of *Foxd1* null embryos to protrude through the cartilage plate, apoptotic cells were labeled by TUNEL analysis. No significant difference in the number of apoptotic pituitary cells was detected in *Foxd1* null embryos as compared to wild type littermates (Fig. 5A–F). This suggests that the pituitary/cartilage dysmorphology in *Foxd1* null embryos is not due to a failure in apoptosis.

**Figure 6:**
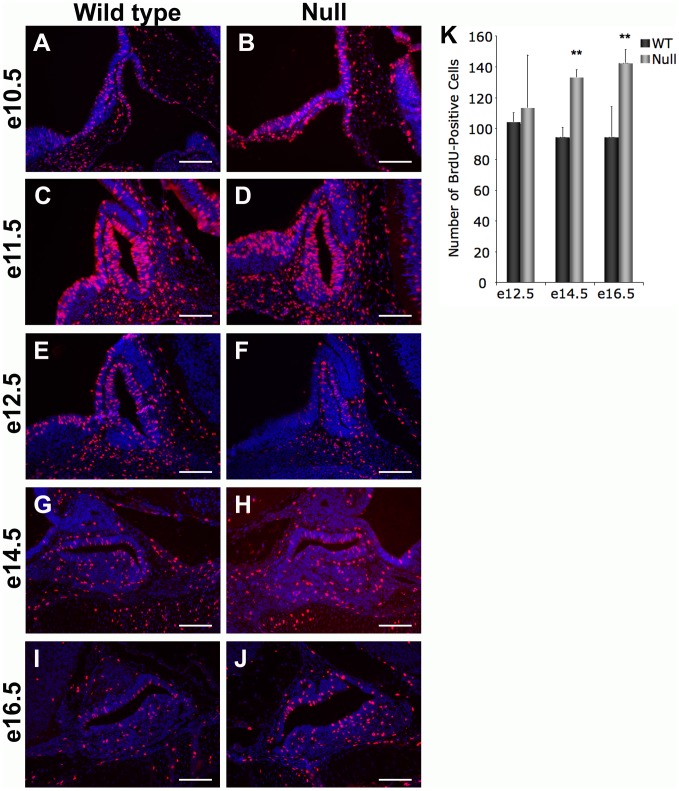
Pituitary cell proliferation is increased in *Foxd1^LacZ/LacZ^* embryos. (A–J) Actively dividing cells were labeled with bromodeoxyuridine (BrdU). Immunohistochemistry was performed to detect BrdU in mid-sagittal sections from *Foxd1^LacZ/LacZ^* embryos and wild type littermates. (K) The number of BrdU-positive cells was manually counted in pituitary sections from *Foxd1^LacZ/LacZ^* embryos and wild type littermates. The number of BrdU-positive cells is significantly higher in *Foxd1^LacZ/LacZ^* embryonic pituitaries at e14.5 and e16.5 as compared to wild type littermates (P<0.01).

Excessive pituitary cell proliferation can cause the pituitary gland to increase in size and protrude through the cartilage plate. Actively dividing cells were labeled in *Foxd1* wild type and null embryos from e10.5 through e18.5 with the thymidine analog bromodeoxyuridine (BrdU) (Fig. 6A–J). The number of BrdU-positive pituitary cells was counted manually. The number of proliferating pituitary cells is significantly increased in *Foxd1^LacZ/LacZ^* embryos at e14.5 and e16.5 (Fig. 6K).

### LHX3

**Figure 7:**
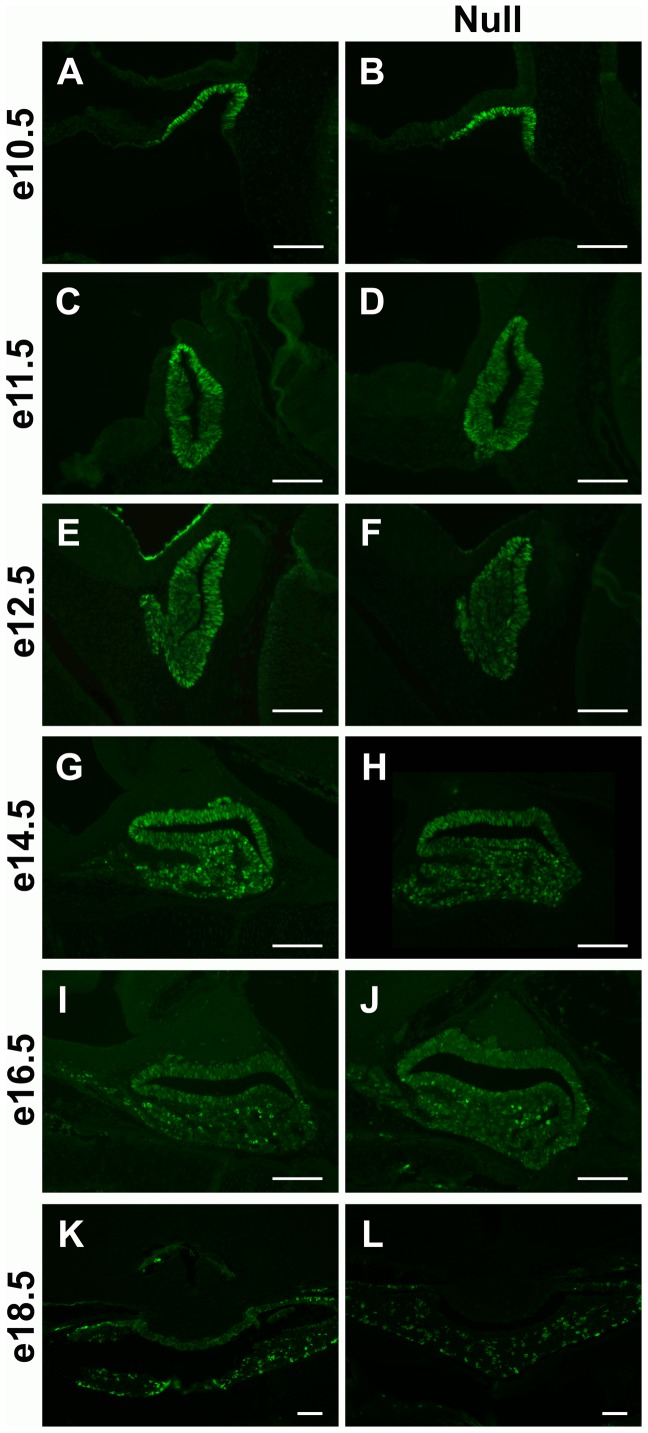
LHX3 patterns are unchanged in *Foxd1^LacZ/LacZ^* embryos. Immunohistochemistry was performed to observe LHX3 patterns in mid-sagittal (A–J) and coronal (K–L) sections of embryonic pituitary glands from *Foxd1^LacZ/LacZ^* embryos and wild type littermates. No difference in LHX3 protein distribution patterns was observed. Pictures were taken at 200X (A–J) or 100X (K–L). Scale bars represent 100 µm.

Fibroblast growth factor (FGF) signaling is essential for normal pituitary development. Pituitary expression of the LIM homeodomain factor, *Lhx3*, is regulated by FGF and loss of *Lhx3* results in a decrease in LH production [31], [32], [33], [34]. To determine if the expression patterns for LHX3 are altered in the developing pituitary in the absence of *Foxd1*, immunohistochemistry for LHX3 was performed. These studies indicate that LHX3 immunoreactivity is not different in *Foxd1* null embryos as compared to wild type littermates (Fig. 7A–L).

## Discussion

Forkhead transcription factors are important for diverse physiological functions including embryonic development of almost every type of tissue [35], [36], [37], [38]. In order to identify genes that contribute to pituitary development and function, we have turned to the forkhead family. *Foxd1* was identified in an embryonic pituitary expression library [26]. However, when *Foxd1* expression was analyzed by β-galactosidase staining of *Foxd1^+/Lacz^* embryos, it was discovered that *Foxd1* is expressed in the mesenchyme surrounding the pituitary, but not in the pituitary itself, suggesting that when dissecting pituitaries from e14.5 mouse embryos, some mesenchyme was obtained as well. The mesenchyme surrounding the developing pituitary gland is a rich source of signaling factors that are essential for normal pituitary development. *Bmp2* is expressed in the mesenchyme ventral and rostral to Rathke’s pouch at e12.5 and e14.5 and is believed to be important for inducing proliferation and differentiation of ventral cell types [5], [39]. The BMP inhibitors chordin and noggin are expressed in the mesenchyme caudally and ventrally, respectively, to the pituitary by approximately e12.5 [5], [27], [39]. FOXD1 is present in the mesenchyme ventral and caudal to the putative pituitary gland from e12.5 and continuing through development. This expression pattern places FOXD1 in a region where it could affect several signaling factors that are important for pituitary development.

Expression of *Lhb* was significantly reduced in *Foxd1* mutant embryos as compared to wild type littermates. These data demonstrate that *Foxd1* is indirectly required for *Lhb* expression in gonadotrope cells. Because *Foxd1* is not expressed in the developing pituitary gland, but is present in the mesenchyme surrounding the developing pituitary, the reduction in *Lhb* expression may be due to the loss of signaling factors from the mesenchyme surrounding the pituitary gland. Signals from outside of the pituitary are important for proper specification of pituitary cells [34]. FGFs from the infundibulum can increase immunorectivity for ACTH and decrease immunoreactivity for the LIM homeodomain factor, ISL1, as well as for αGSU [34]. In contrast, BMPs from the ventral juxtapituitary mesenchyme increases immunoreactivity for αGSU and decreases immunoreactivity for ACTH [34]. In embryos null for noggin, a BMP2 and 4 antagonist, a secondary pituitary is sometimes induced, however in the primary pituitary all hormones are produced normally [27]. The BMP antagonist, chordin, is expressed in the caudal mesenchyme adjacent to Rathke’s pouch at e12.0 and may be important for counteracting BMP2 signals [5]. It may be that FOXD1 is important for production of one or more of these signaling factors and that loss of FOXD1, and ultimately of certain signals from the mesenchyme, disrupts *Lhb* expression.

Another possibility is that loss of *Foxd1* in the mesenchyme surrounding the hypothalamus affects hypothalamic function or that GnRH neuronal migration is abnormal. In mouse embryos lacking *Foxd1* retinal ganglion cell axons that form the optic chiasm aberrantly project contralaterally [22]. Thus, FOXD1 appears to be important for proper neuronal migration. *Hpg* mice, which have a mutation of the *Gnrh* gene resulting in a loss of GnRH secretion, exhibit reduced expression of *Lhb* and *Fshb* [40], [41]. In *Foxd1* mice expression of the GnRH-responsive genes *Fshb* and *Cga* is normal. Thus, it seems unlikely that GnRH stimulation of gonadotrope cells is altered in *Foxd1* mutants.

Apoptosis is an important process in pituitary development, allowing the oral ectoderm that will form the pituitary gland to separate from the oral ectoderm that will form the lining of the mouth with a cartilage plate forming between. *Foxd1* mutants exhibit normal apoptosis, however abnormal apoptosis has been observed with other mutations [27], [31], [42], [43]. The pituitary fails to separate from the lining of the oral cavity in the absence of *Lhx3* expression, however it has not been determined if a failure in apoptosis during early development is responsible [31]. Increased apoptosis later in development does contribute to the hypopituitarism observed in *Lhx3* null embryos [42], [43]. In the absence of the BMP inhibitor, noggin, apoptosis in Rathke’s pouch is decreased and the pituitary fails to separate from the oral ectoderm, disrupting formation of the cartilage plate [27].

Proliferation is an essential process in organogenesis. During pituitary development, proliferation occurs in Rathke’s pouch, but not in the rostral tip of the pituitary gland [6]. Loss of TCF4, which mediates WNT signaling, causes pituitary cell hyperplasia, leading to a disruption of the cartilage plate ventral to the pituitary gland [44], [45]. The number of proliferating pituitary cells was increased at e14.5 and e16.6 in embryos lacking *Foxd1*, suggesting that *Foxd1* is important for regulating proliferation in the developing pituitary gland. This increase in pituitary cell proliferation may contribute to the break in the cartilage plate ventral to the pituitary gland as seen in *Tcf4* mutants.

There is much to be learned about how signaling factors from the juxtapitutiary mesenchyme regulate pituitary hormone production. The data described in this manuscript demonstrate that *Foxd1* indirectly contributes to normal expression of *Lhb*, but not the gonadotropin genes *Fshb* and *Cga*. In the absence of *Foxd1*, pituitary cell proliferation is increased and the cartilage plate ventral to the pituitary fails to fuse. These studies provide important clues as to the role of FOXD1 and the juxtapituitary mesenchyme during pituitary development and for pituitary function.
